# Ammonium Is Toxic for Aging Yeast Cells, Inducing Death and Shortening of the Chronological Lifespan

**DOI:** 10.1371/journal.pone.0037090

**Published:** 2012-05-15

**Authors:** Júlia Santos, Maria João Sousa, Cecília Leão

**Affiliations:** 1 Life and Health Sciences Research Institute (ICVS), School of Health Sciences, University of Minho, Braga, Portugal; 2 ICVS/3B's - PT Government Associate Laboratory, Braga/Guimarães, Portugal; 3 Molecular and Environmental Research Centre (CBMA)/Department of Biology, University of Minho, Braga, Portugal; University of Washington, United States of America

## Abstract

Here we show that in aging *Saccharomyces cerevisiae* (budding yeast) cells, NH_4_^+^ induces cell death associated with shortening of chronological life span. This effect is positively correlated with the concentration of NH_4_^+^ added to the culture medium and is particularly evident when cells are starved for auxotrophy-complementing amino acids. NH_4_^+^-induced cell death is accompanied by an initial small increase of apoptotic cells followed by extensive necrosis. Autophagy is inhibited by NH_4_^+^, but this does not cause a decrease in cell viability. We propose that the toxic effects of NH_4_^+^ are mediated by activation of PKA and TOR and inhibition of Sch9p. Our data show that NH_4_^+^ induces cell death in aging cultures through the regulation of evolutionary conserved pathways. They may also provide new insights into longevity regulation in multicellular organisms and increase our understanding of human disorders such as hyperammonemia as well as effects of amino acid deprivation employed as a therapeutic strategy.

## Introduction

Starvation of an exponentially growing yeast culture for a given nutrient usually results in the growth arrest of cells in the culture in an unbudded state as they exit the cell cycle. Under extreme starvation conditions such as culturing in water, cells can attain a quiescent state and are able to survive for long periods. The time cells survive in this non-dividing state, known as chronological life span (CLS), is dependent on pre-culture conditions, reaching a maximum for cells grown on respiratory carbon sources and allowed to reach stationary phase [1]. Several nutrient signaling pathways have been implicated in the regulation of yeast CLS, mainly TOR (target of rapamycin), PKA (protein kinase A) and Sch9p [2]. In *Saccharomyces cerevisiae*, TOR signaling responds to nitrogen and possibly to carbon sources. This pathway controls cell growth by activating anabolic processes and inhibiting catabolic processes and mRNA degradation [3]–[5]. Inactivation of TORC1 (TOR complex 1) or other members of the TOR pathway is accompanied by phenotypic changes characteristic of starved cells, protects against stress, and leads to extension of the longevity of non-dividing yeast [6], [7].

The PKA pathway also plays a major role in the control of metabolism, stress resistance, cell cycle, growth, and transcription. It is highly regulated by the nutrient composition of the medium, in particular by the presence of a rapidly fermentable sugar and other essential nutrients such as amino acids and phosphate or ammonium [8], [9]. Addition of a rapidly fermentable sugar triggers activation of adenylate cyclase (Cyr1p) and a rapid increase in cAMP levels. This increase boosts the activity of the cAMP-dependent PKA by displacing the regulatory subunit Bcy1p from the catalytic subunits Tpk1p, Tpk2p and Tpk3p. PKA affects several downstream targets, thereby allowing cells to make the necessary adaptations for fermentative growth. These adaptations include upregulation of glycolysis, stimulation of cell growth and cell cycle progression, downregulation of stress resistance and gluconeogenesis, and mobilization of the reserve carbohydrate glycogen and the stress protector trehalose [10], [11]. Down-regulation of the PKA pathway by starvation of an essential nutrient causes growth arrest and subsequent entrance into G0. Cells in G0 acquire a variety of characteristics such as accumulation of the carbohydrates trehalose and glycogen, induction of stress-responsive element- and postdiauxic shift-controlled genes, induction of autophagy and increased stress resistance [8], [12]. Mutations in components of PKA pathway confer chronological life span extension [13].

The protein kinase Sch9 also plays an important role in nutrient-mediated signaling. It acts in parallel with the PKA pathway and is directly phophorylated by TORC1, mediating many of the TORC1-regulated processes [10], [14]. Recent studies revealed that Sch9p also acts independently of TORC1, and can even exert opposite effects to TORC1 in the adaptation to stressful conditions [15].

It has previously been shown that, in the absence of other nutrients, adding glucose to cells suspended in water can cause cells to exit the quiescent state and commit to an apoptotic cell death program that includes production of reactive oxygen species (ROS), RNA and DNA degradation, membrane damage, nucleus fragmentation and cell shrinkage [16]. Chronological aging of yeast cells in medium also results in a loss of viability with increasing time accompanied by morphological and biochemical characteristics of both apoptosis and necrosis [17], [18]. In the present work, we aimed to identify other nutrient signals that could induce cell death of chronological aging yeasts and the signaling pathways involved. Ammonium (NH_4_^+^) is a nitrogen source commonly used for yeast growth and it is usually not toxic. Production of ammonia in yeast colonies has even been described as a mechanism of protection from cell death during colony development [19]. To our knowledge, only one report in the literature refers to NH_4_^+^ toxicity in yeast, which was observed in steady-state chemostat cultures limited for potassium [20].

We have found that decreasing the concentration of NH_4_^+^ in the culture medium increases yeast CLS. Furthermore, we have extensively characterized for the first time a cell death process induced by NH_4_^+^ in yeast cells. NH_4_^+^ induced loss of cell viability in aging *S. cerevisiae* cultures either in nutrient-depleted culture medium or upon transfer to water with NH_4_^+^. This effect was particularly significant for cells starved for auxotrophic-complementing amino acids, but not completely starved for nitrogen. We also determined that activation of PKA stimulated NH_4_^+^ - induced cell death, consistent with the observation that deficiency in upstream components of the cAMP PKA pathway partially reverted the toxic effect of ammonium. Deletion of *TOR1* also significantly rescued NH_4_^+^ - induced cell death and decreased PKA activation. In contrast, *SCH9* deletion abolished PKA activation in response to NH_4_^+^ but did not revert the decrease in cell viability. This indicates that PKA inactivation cannot protect cells from NH_4_^+^ - induced cell death in the absence of Sch9p, suggesting a potential role of Sch9p in cell survival.

NH_4_^+^-induced cell death has been implicated in a number of different human disorders that are accompanied by hyperammonemia [21]. However, the precise molecular mechanisms triggering NH_4_^+^-induced cell death in these disorders are not known. In addition, deprivation of essential amino acids has been employed as a strategy in cancer therapy but resistance has often been found [22]. Our results enhance our understanding of longevity regulation in multicellular organisms. They also suggest that *S. cerevisiae* might serve as a useful model for the identification of signaling pathways and new therapeutic targets for the referred human disorders.

## Results

### NH_4_^+^ causes loss of survival in chronologically aged yeast cells

**Figure 1:**
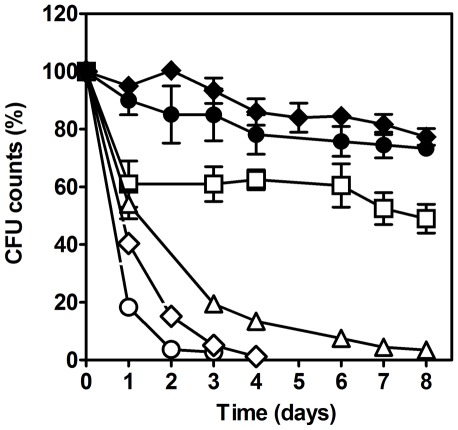
Ammonium stimulates CLS shortening. Survival of *S. cerevisiae* stationary phase cells grown in media supplemented with low (open symbols) and high (dark symbols) concentrations of auxotrophy-complementing amino acid, and supplemented with 0.01% (□); 0.1% (▵); 0.5% (⋄,⧫) or 1% (○,•) ammonium sulphate. In all the cultures, starting cell density was about 3.8×10 ^7^ cells/ml. Values are means ± SEM (n = 3). *P*<0.001. Statistical analysis was performed by two-way ANOVA.

The chronological life span (CLS) of *S. cerevisiae* is strongly affected by the concentration of the auxotrophy-complementing amino acid in the medium. Cells of the auxotrophic *S. cerevisiae* strain BY4742 cultured with an insufficient supply of essential amino acids display reduced lifespan compared with cells grown with increased amino acid supplementation in the medium [23]. In the present work we observed that BY4742 cells grown with insufficient supply of amino acids grow less than those without this restriction, and neither glucose (as previously reported [23]) nor NH_4_^+^ are completely depleted (Fig. S1A). We first asked whether manipulating the ammonium concentration in the culture medium might affect CLS as previously described for glucose [24]. Reducing the starting concentration of (NH_4_)_2_SO_4_ in the medium five- or fifty-fold (from 0.5% to 0.1 and 0.01%, respectively) improved the survival of chronological aging cells cultured with amino acid restriction (Figure 1). In contrast, when the initial (NH_4_)_2_SO_4_ concentration in the culture medium, either with or without restriction of amino acids, was increased to 1%, there was a decrease in cell survival, although loss of cell viability was much faster for cells grown with amino acid restriction (Figure 1).

**Figure 2:**
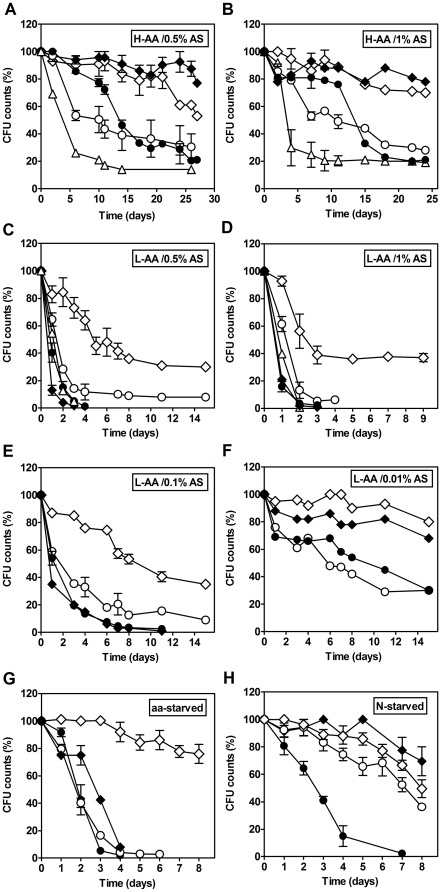
Ammonium stimulates cell death of *S. cerevisiae* associated with a shortening of the CLS. **A, B, C, D, E and F.** Survival of *S. cerevisiae* stationary phase cells grown in media supplemented with low (L-AA) and high (H-AA) concentrations of auxotrophy-complementing amino acid, and supplemented with (F) 0.01%; (E) 0.1%; (A, C) 0.5% or (B, D) 1% ammonium sulphate (AS). After 72 hours of growth, cells were transferred to: (⋄) water (pH 7.0); (○) water with 0.5% (NH_4_)_2_SO_4_ (pH 7.0); (▵) water with 1% (NH_4_)_2_SO_4_ (pH 7.0); (•) exhausted medium; (⧫) exhausted medium (pH 7.0). Values are means ± SEM (n = 3–5). **G and H** - Survival of (H) nitrogen starved cells (N-) or (G) amino acid-starved cells (aa-), after transfer to: (⋄) water (pH 7.0); (○) water with 0.5% (NH_4_)_2_SO_4_ (pH 7.0); (•) starvation medium; (⧫) starvation medium (pH 7.0). In all the cultures, starting cell density was about 3.8×10 ^7^ cells/ml. Values are means ± SEM (n = 8). *P*<0.001 (aa-starved H_2_O *vs* aa-starved 0.5% (NH_4_)_2_SO_4_). Statistical analysis was performed by two-way ANOVA.

We sought additional insights into this phenomena by asking whether increased NH_4_^+^ could account for the loss of cell viability, as reported in earlier studies [16] which showed that adding glucose to yeast suspensions in water also causes cells to rapidly die. Cells were grown in SC 2% glucose plus 0.5% (NH_4_)_2_SO_4_, with or without amino acid restriction in the medium for 72 hours and then transferred to water without NH_4_^+^ (pH 7.0), water with NH_4_^+^ (pH 7.0), or to the depleted medium as a control. It has been shown that medium acidification limits survival of yeast cells during chronological aging in SC medium and that the longer survival observed in water can be, at least in part, attributed to the differences in pH [25]–[27]. To assess whether acidification could play a role in the NH_4_^+^ -induced loss of cell viability, we measured cell survival in media without adjusting pH (pH 2.6–2.9 due to culture acidification) or adjusted to pH 7.0 (see schematic of methodology in Figure S2). When cells were transferred to water or to depleted medium that was either adjusted or not adjusted to pH 7.0, there was no significant pH variation during the entire experiment. As shown in Figure 2, cells grown with or without amino acid deprivation exhibited a longer CLS after they were transferred to water compared to cells transferred to depleted culture medium that maintained a pH of 2.6–2.9, although loss of cell viability again occurred much faster for cells grown with amino acid restriction. In all cases, addition of NH_4_^+^ to water reduced cell survival in proportion to its concentration, mimicking its effect in the depleted media. Furthermore, the NH_4_^+^-induced reduction in CLS observed in water positively correlated with the concentration of NH_4_^+^ in the growth medium, which indicates that culture conditions pre-determined the cellular response to NH_4_^+^. Furthermore, transferring cells cultured with insufficient supply of amino acids with 1%, 0.5% or 0.1% (NH_4_)_2_SO_4_ to the respective exhausted medium adjusted to pH 7.0 did not lead to a significant difference in CLS relative to the CLS of cells in the exhausted acidic medium (Figure 2C, 2D and 2E). In contrast, cells cultured under amino acid restriction conditions with the lowest (NH_4_)_2_SO_4_ concentration (0.01%) after transfer to the respective exhausted medium adjusted to pH 7.0 exhibited an extended CLS (Figure 2F). Similar results were obtained with cells cultured without amino acid restriction (Figure 2A and 2B), which is consistent with results previously described for similar conditions [26].

To further explore the toxic effects of NH_4_^+^ during chronological aging under amino acid restriction conditions, a conventional nitrogen starvation protocol [28] was adapted to accommodate the following conditions in SC glucose starvation medium: i) lack of the auxotrophy-complementing amino acids and presence of NH_4_^+^ (aa-starved cells) or ii) lack of the auxotrophy-complementing amino acids and of NH_4_^+^ (N-starved cells). Cells were grown to mid exponential phase in SC medium with 2% glucose and then starved for 24 hours in both types of starvation media. Cells were subsequently transferred to water (pH 7.0), with and without NH_4_^+^ or to the respective 24 hour starvation medium (final pH 2.7–2.9) that was or was not adjusted to pH 7.0 (see scheme of methodology in Figure S2). The initial pH did not significantly change during the assay, except for cells transferred to starvation media at pH 7.0, which reached a final pH around 5.0.

Both aa-starved and N-starved cells survived for a longer period of time in water relative to those in starvation medium (pH 2.7–2.9). Addition of NH_4_^+^ to water induced a rapid loss of cell viability and shortening of CLS for aa-starved cells (Figure 2G and 2H). Cells cultured in starvation medium that was adjusted to pH of 7.0 also exhibited a rapid decrease in cell viability, indicating that the NH_4_^+^ effect is not due to the acidification of the medium. In contrast to aa-starved cells, N-starved cells survived for a longer period when transferred to the starvation medium adjusted to pH 7.0. To eliminate the possibility that the reduced survival of aa-starved cells induced by the addition of (NH_4_)_2_SO_4_ to water was due to sulphate and not to ammonium, the same experiment was performed in water to which NH_4_OH was added instead of (NH_4_)_2_SO_4_, and similar results were obtained (Figure S1B).

As discussed in the Introduction, NH_4_^+^ toxicity was previously described in steady-state chemostat cultures of yeast under limiting potassium concentration [20]. To determine if the ammonium toxicity we observed in our experiments depends on potassium concentration, we repeated our experiments after adding potassium to water at a concentration that according to this earlier study abolished NH_4_^+^ toxicity. In fact, addition of potassium did not alter the NH_4_^+^ -induced loss of cell viability (Figure S1C).

Taken together, our results (summarized in Table 1) suggest that (i) NH_4_^+^ in the culture medium has a substantial concentration-dependent inhibitory effect on CLS indicated by a significant increase in cell survival when the starting NH_4_^+^ concentration in the medium is reduced; (ii) the CLS of cells cultured to stationary phase with amino acid restriction or starved for auxotrophy-complementing amino acids and subsequently transferred to water is significantly shortened by the addition of NH_4_^+^ and (iii) acidification of the medium does not promote the observed decrease in cell survival, in contrast to what is observed at the lowest NH_4_^+^ concentration and in cells grown without amino acid restriction.

**Table 1:** Values of Area under the survival curve (AUC) of strain BY4742 cultured in different medium composition.

		Cell culture or pre-incubation conditions							
		High-AA		Low-AA				aa-starved	N-starved
		(NH_4_)_2_SO_4_0.5%	(NH_4_)_2_SO_4_1%	(NH_4_)_2_SO_4_0.01%	(NH_4_)_2_SO_4_0.1%	(NH_4_)_2_SO_4_0.5%	(NH_4_)_2_SO_4_1%	(NH_4_)_2_SO_4_0.5%	
**Aging assay in:**	MediumSc	1205±13	1175±2	805±47	207±12	115±5	71±5	186±7	272±3
	MediumSc pH7	1439±33	1202±5	1214±2	175±5	74±9	74±2	249±3	725±1
	H_2_O	1375±87	1347±29	1390±5	916±25	745±42	446±44	723±10	679±2
	(NH_4_)_2_SO_4_0.5%	923±77	951±5	706±23	385±20	271±13	133±12	188±10	588±16
	(NH_4_)_2_SO_4_1%	535±2	552±102	n.d.	n.d.	120±10	90±7	n.d.	n.d.

n.d. – not determined. Cells were grown in SC media supplemented with low (Low-AA) or high (High-AA) concentrations of auxotrophy-complementing amino acid and with 0.01%; 0.1%; 0.5% or 1% (NH_4_)_2_SO_4_ for 72 hours; or cells were grown in SC media until O.D. 1–1.5, harvested and resuspended in Nitrogen-starvation medium (N-) or in amino acid-starvation medium (aa-) for 24 hours. The aging assays were performed by resuspending cells from the different culture conditions in their respective exhausted medium, exhausted medium pH 7, or in 0.5 and 1% (NH_4_)_2_SO_4_, pH 7.

Consequently, in subsequent experiments we employed aa-starved cells to address the mechanisms underlying cell death induced by NH_4_^+^.

### NH_4_^+^ induces apoptosis and necrosis in association with the reduction in CLS in amino acid starved yeast cells

**Figure 3:**
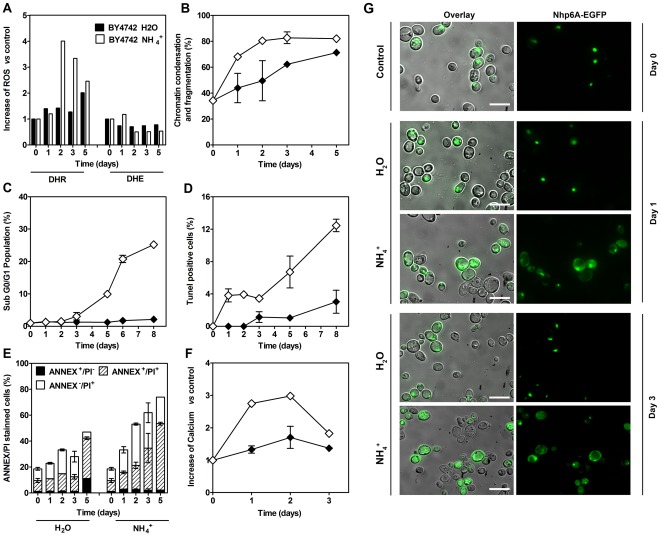
Ammonium-induced cell death was accompanied by an initial small increase of apoptotic cells followed by extensive necrosis. Cell death markers measurements in aa-starved cells of *S. cerevisiae,* upon transfer to: (⧫) water (pH 7.0) or (⋄) water with 0.5% (NH_4_)_2_SO_4_ (pH 7.0). (A) ROS accumulation, (B) chromatin condensation and fragmentation, (C) appearance of Sub-G0/G1 peak, (D) TUNEL staining, (E) Annexin/PI positive staining and (F) calcium accumulation. (G) Fluorescence microscopy of aa-starved cells (day 0, 1 and 3) expressing Nhp6A–EGFP, upon transfer to water (pH 7.0) or water with 0.5% (NH_4_)_2_SO_4_ (pH 7.0). Scale bars, 10 µm. In all the cultures, starting cell density was about 3.8×10^7^ cells/ml. Values are means ± SEM (n = 3). H_2_O *vs* 0.5% (NH_4_)_2_SO_4_: (A) *P*<0.001; (C) *P*<0.001; (D) *P*<0.01; (E) *P*<0.001; (F) *P*<0.01. Statistical analysis was performed by two-way ANOVA.

To determine the mechanism by which cell death occurs in association with the reduction in CLS induced by NH_4_^+^, several standard markers of cell death were examined in aa-starved cells transferred to water alone or to water containing NH_4_^+^ after the pH was adjusted to 7.0 in both cases. Increased ROS accumulation is a common event in many cell death scenarios, both apoptotic and necrotic [29]–[31]. We measured the accumulation of reactive oxygen species (ROS) using the fluorescent probe dihydrorhodamine 123 (DHR, which preferentially detects H_2_O_2_). DHR signals increased with time either in the absence or presence of NH_4_^+^, but this increase occurred more rapidly in cells incubated with NH_4_^+^, peaking at day 2 (Figure 3A). In contrast, levels of ROS detected using dihydroethidium (DHE, which preferentially detects O_2_^−^), were not significantly different in the absence or presence of NH_4_^+^. The shorter CLS induced by NH_4_^+^ was also accompanied by an increase in the number of cells exhibiting chromatin condensation and nuclear fragmentation (Figure 3B) and by the emergence of a population of cells with a sub G0/G1 content of DNA that increased over time (Figure 3C and S3A). Incubation with NH_4_^+^ also resulted in an increase in TUNEL positive cells, although this occurred in a relatively small percentage of the total population (Figure 3D). Furthermore, staining with annexin V and PI was used to identify apoptotic and necrotic cells [30]. In this double staining approach, annexin V binds phosphatidylserine of the plasma membrane whereas PI, being a membrane-impermeable stain, assesses loss in membrane integrity. Annexin V+/PI− staining shows cells with phosphatidylserine exposed on the outer surface of the plasma membrane in the absence of a loss in membrane integrity and therefore cells are considered apoptotic, while PI+ cells are necrotic. Cells transferred to water containing NH_4_^+^ exhibited a small increase in Annexin V staining in the absence of PI staining during the first few days (Figure 3E). However, after day 2 these cells exhibited extensive permeabilization of the plasma membrane evidenced by PI staining, which indicates they were mostly undergoing necrosis. Necrosis was confirmed by the observation of the nucleus-cytosolic translocation of Nhp6Ap (Figure 3G), the yeast homologue of human chromatin bound non-histone protein HMGB1 (high mobility group Box 1) whose nuclear release is considered a marker of necrosis [17]. Also consistent with necrosis, we observed a significant decrease in ATP content in these cells beginning on the first day of assays (Fig. S3B) which may have limited energy consuming apoptotic processes. Furthermore, NH_4_^+^-induced cell death was not prevented by cycloheximide (Figure S4D), indicating that death is not dependent on *de novo* protein synthesis. Together these data point to an initial apoptotic cell death induced by NH_4_^+^ followed by a rapid secondary necrosis.

To clarify the mechanism(s) of cell death induced by NH_4_^+^, we employed strains from which genes coding for the yeast metacaspase (Yca1p), apoptosis inducing factor (Aif1p), mitochondrial cyclophylin (Cpr3p) and calpain (Rim13p) had been deleted. Loss of cell viability induced by NH_4_^+^ in aa-starved cells in water was not altered by deletion of either *YCA1* or *AIF1* (Figure S5A and S5B). Therefore, cell death does not depend on Yca1p or Aif1p, which are key factors in several yeast apoptotic processes [32], [33]. Strains deleted in *RIM13* and *CPR3* coding for the yeast orthologs of mammalian proteins previously associated with necrotic phenotypes [29] displayed loss of cell viability induced by NH_4_^+^ in water similar (*cpr3Δ*) or higher (*rim13Δ*), when compared to wild type strain (Figure S5C and S5D), indicating that those genes are not associated with the NH_4_^+^ sensitivity phenotype. In agreement with the results obtained with the *cpr3Δ* mutant, loss of cell viability induced by NH_4_^+^ in aa-starved wild type cells in water was also not altered by simultaneous incubation with cyclosporine, an inhibitor of mitochondrial cyclophylin (Figure S5E). We observed an increase in death induced by NH_4_^+^ in the *rim13Δ* mutant, suggesting that instead of mediating cell death, Rim13p, belonging to the calpain family of cysteine protease that are activated by Ca^2+^ [34], may protect against cell death. Consistent with the involvement of calpain activity, an increase in the intracellular calcium concentration was observed in the presence of NH_4_^+^ (Figure 3F).

In summary, although cell death induced by NH_4_^+^ in aa-starved cells was accompanied by chromatin condensation and DNA fragmentation, both of which suggest an apoptotic process, the subsequent loss of membrane integrity points to accelerated necrosis at later times that may be partially rescued by calpain.

### Autophagy is not a key player in NH_4_^+^-induced cell death

**Figure 4:**
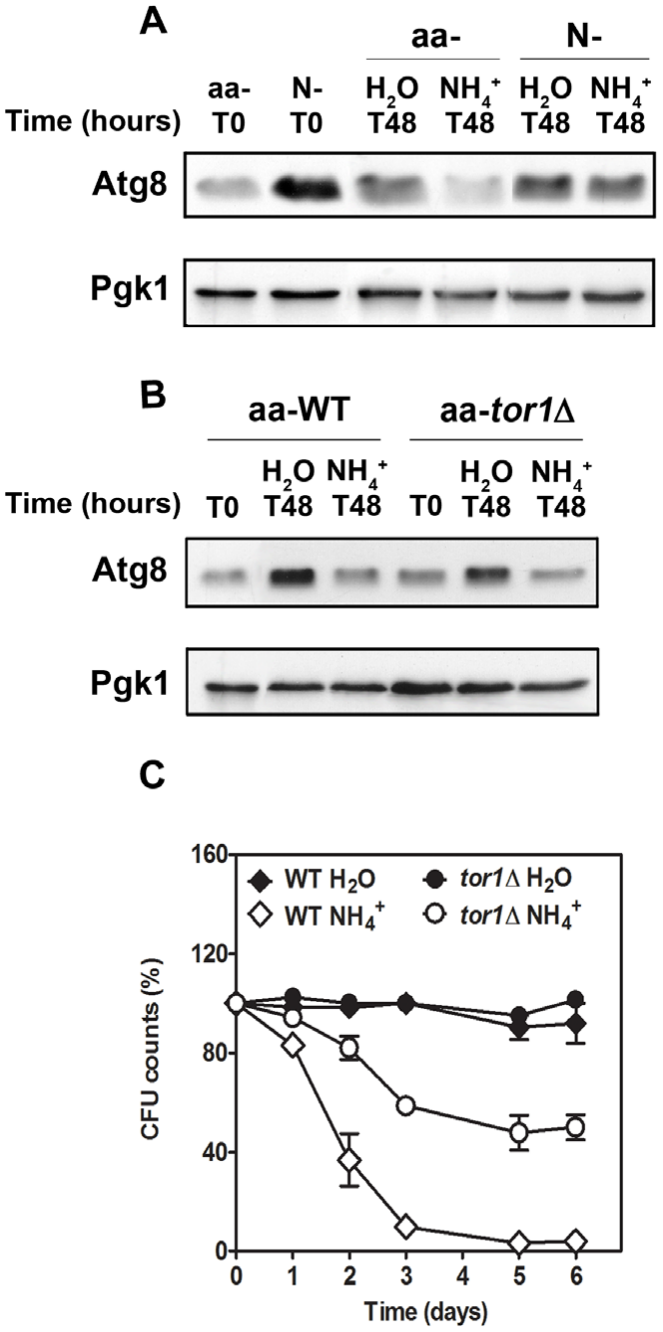
Ammonium inhibits autophagy induction in aa-starved cells of *S. cerevisiae*. Western-blot analysis of Atg8p levels present in: (A) wild-type (WT) aa-starved or N-starved cells, upon transfer to water or water with 0.5% (NH_4_)_2_SO_4_; and in (B) WT and *tor1Δ* aa-starved cells, upon transfer to water or water with 0.5% (NH_4_)_2_SO_4_. (C) Survival of aa-starved cells of wild-type and *tor1*Δ mutant, upon transfer to water or water with 0.5% (NH_4_)_2_SO_4_. In all the cultures, starting cell density was about 3.8×10^7^ cells/ml and the initial pH was adjusted to 7.0. Values are means ± SEM (n = 3–4). (C) *P*<0.001 (H_2_O *vs* 0.5% (NH_4_)_2_SO_4_). Statistical analysis was performed by two-way ANOVA.

Autophagy is regulated by nitrogen availability via the major nutrient signalling pathways, which also regulate CLS [6], [35]. Therefore, we asked whether autophagy might be required for the NH_4_^+^-induced decrease in CLS. *ATG8* codes for a protein essential for autophagosome assembly and its expression is up-regulated by nitrogen starvation shortly after autophagy induction [36]. Thus, we monitored Atg8p levels in cells starved for amino acids (aa- starved cells), before and after transfer to water with or without NH_4_^+^ (Figure 4A). As expected, autophagy was induced in control cells completely starved for nitrogen (N-starved cells). Autophagy was not induced, however, in aa-starved cells before they were transferred to water, although autophagy was detected in both aa- and control N-starved cells after transfer to water in the absence of NH_4_^+^. Importantly, the presence of NH_4_^+^ in water inhibited induction of autophagy in aa-starved cells but not in control N-starved cells.

To evaluate the impact of inhibiting autophagy on cell viability, we used a mutant in the TOR pathway (*tor1Δ*). Tor1p associates with Tor2p and three other proteins to form the TORC1 complex, which negatively regulates autophagy [10]. The *tor1Δ* mutant also did not exhibit autophagy either after amino acid starvation or upon transfer to water with NH_4_^+^ (Figure 4B). However, there was a significant reduction in NH_4_^+^ toxicity in this mutant (Figure 4C), thus excluding inhibition of autophagy as a causal factor in NH_4_^+^-induced cell death. To address this point further, we employed wortmannin, an inhibitor of PI3-kinases that blocks autophagy, as well as a mutant deficient for *ATG8* (Figure S4). *atg8Δ* aa-starved cells in water with NH_4_^+^ displayed loss of cell viability similar to that of wild type (*WT*) cells (Figure S4A). Addition of wortmannin to aa-starved *WT* cells incubated in water with NH_4_^+^ also had no effect in cell survival (Figure S4B). Furthermore, NH_4_^+^ -induced cell death was not observed in *atg8Δ* N-starved cells (Figure S4C). These results indicate that although NH_4_^+^ inhibits autophagy, autophagy inhibition is not the cause of the NH_4_^+^ -induced cell death observed in aa-starved cells.

### PKA and TOR regulate the ammonium-induced reduction in the CLS of amino acid-starved yeast cells

**Figure 5:**
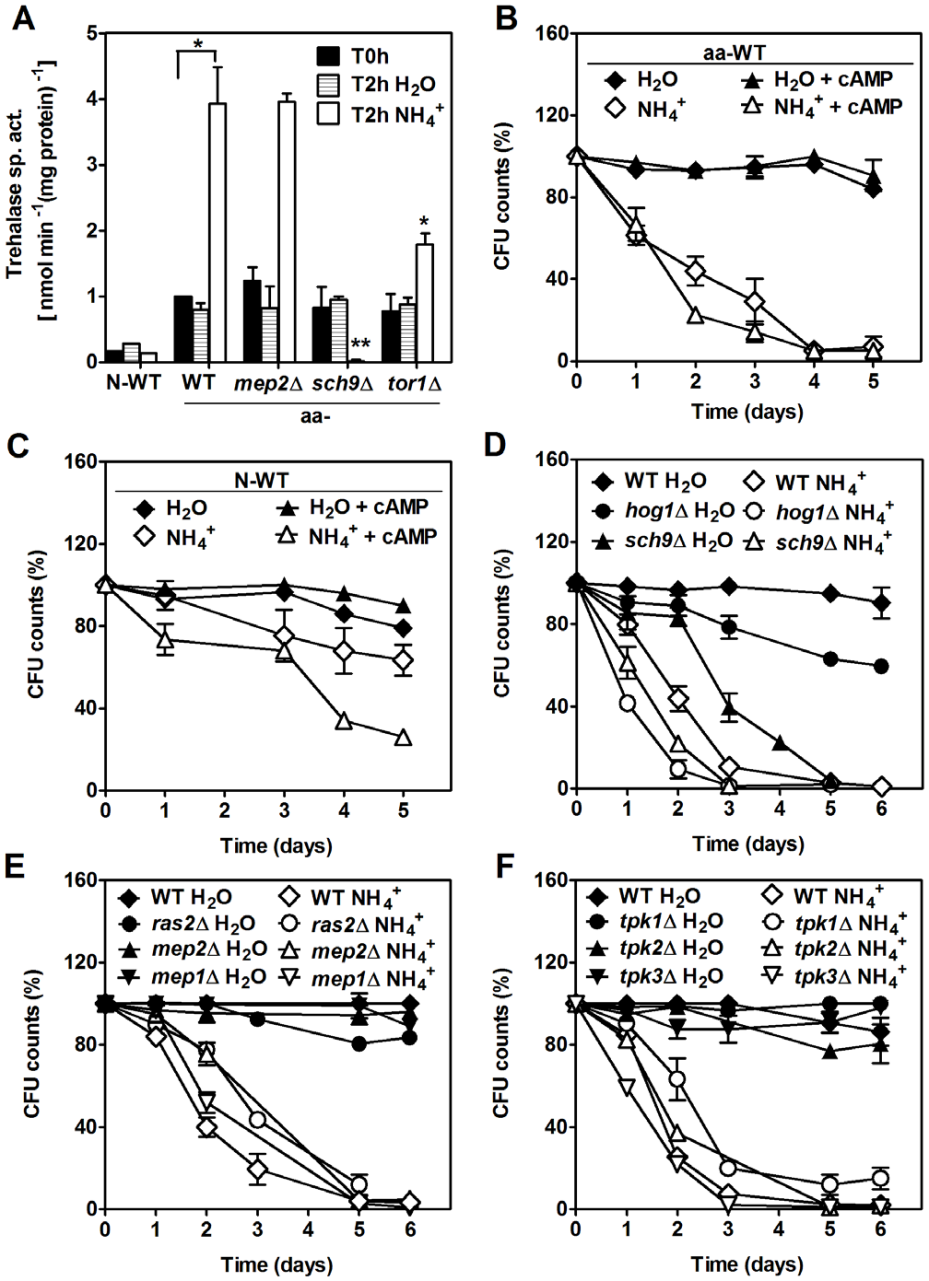
Ammonium reduces CLS of *S. cerevisiae* through the regulation of both PKA and Sch9 activities. (A) Trehalase activity of wild-type (WT) N-starved cells and WT and mutant (*mep2Δ*, *sch9Δ* and *tor1Δ*) aa-starved cells, before transferred to water (T0h) and after 2 hours in water (T2h H_2_O) or water with 0.5% (NH_4_)_2_SO_4_ (T2h NH_4_^+^). Survival of (B) wild-type aa-starved cells or (C) N-starved cells, after transfer to: (⧫) water (pH 7.0); (⋄) water with 0.5% (NH_4_)_2_SO_4_ (pH 7.0); (▴) water (pH 7.0) supplemented with cAMP (4 mM); (▵) water with 0.5% (NH_4_)_2_SO_4_ (pH 7.0) supplemented with cAMP (4 mM). Survival in water (pH 7.0) or water with 0.5% (NH_4_)_2_SO_4_ (pH 7.0) of aa-starved cells of: (D) WT, *sch9Δ* and *hog1Δ*; (E) WT, *mep1Δ*, *mep2Δ* and *ras2Δ*; (F) WT, *tpkΔ* mutants (*tpk1Δ*, *tpk2Δ* or *tpk3Δ*). In all the cultures, starting cell density was about 3.8×10^7^ cells/ml and the initial pH was adjusted to 7.0. Values are means ± SEM (n = 3–4). (A) *P<0.05 (WT H_2_O *vs* WT 0.5% (NH_4_)_2_SO_4_), (WT 0.5% (NH_4_)_2_SO_4_ *vs tor1Δ* 0.5% (NH_4_)_2_SO_4_); **P<0.01 (WT 0.5% (NH_4_)_2_SO_4_ *vs sch9Δ* 0.5% (NH_4_)_2_SO_4_); (D) *P*<0.05 (WT 0.5% (NH_4_)_2_SO_4_ *vs hog1Δ* 0.5% (NH_4_)_2_SO_4_), *P*<0.001 (WT H_2_O *vs sch9Δ* H_2_O); (E) *P*<0.05 (WT 0.5% (NH_4_)_2_SO_4_ *vs ras2Δ* 0.5% (NH_4_)_2_SO_4_); *P*<0.01 (WT 0.5% (NH_4_)_2_SO_4_ *vs mep2Δ* 0.5% (NH_4_)_2_SO_4_); *P*<0.05 (WT 0.5% (NH_4_)_2_SO_4_ *vs mep1Δ* 0.5% (NH_4_)_2_SO_4_); (F) *P*<0.01 (WT 0.5% (NH_4_)_2_SO_4_ *vs tpk1Δ* 0.5% (NH_4_)_2_SO_4_). Statistical analysis was performed by two-way ANOVA.

CLS is under the control of both TOR, Sch9p and PKA signalling pathways [6]. The absence of autophagy inhibition as a causal factor in NH_4_^+^ -induced cell death led us to hypothesize that NH_4_^+^ toxicity might be mediated by PKA activation instead. Trehalase is a target of PKA regulation and its activity has been extensively used to monitor PKA activation [11]. As shown in Figure 5A, trehalase activity was much higher in aa-starved cells upon transfer to water with NH_4_^+^ than in the same cells without NH_4_^+^ or in N-starved cells (negative control) under both conditions. In support of the hypothesis that activation of PKA increases sensitivity to NH_4_^+^, addition of cAMP increased cell death in the presence of NH_4_^+^ in N-starved cells and had no effect on aa-starved cells, which display high PKA activity in the absence of added cAMP (Figure 5B and 5C). In addition, deletion of *RAS2*, a regulator of PKA activity through the stimulation of cAMP production, caused a partial reversion of the NH_4_^+^ sensitivity phenotype of aa-starved cells (Figure 5E). The NH_4_^+^ permease Mep2 (and Mep1 to a lesser extent) function as sensors for NH_4_^+^-induced activation of PKA, whereas Mep3p, the other member of the family of NH_4_^+^ transporters, does not [28], [37]. As shown in Figure 5E, the *mep2Δ* and *mep1Δ* strains exhibited a decrease in NH_4_^+^-induced death in aa-starved cells, although this decrease was significant only in *mep2Δ*. This is in agreement with the more predominant role of Mep2p in PKA signalling. In order to identify the specificity of the signalling process through PKA, we also tested the effects of deleting the genes that code for the three isoforms of the catalytic subunit of this kinase, *TPK1*, *TPK2* and *TPK3*. Only deletion of *TPK1* caused a significant reversion of the NH_4_^+^-induced decrease of the CLS, whereas no differences were detected for strains deficient in *TPK2* and *TPK3* (Figure 5F).

Sch9p is a protein kinase with high sequence homology to Tpk1, 2, 3 kinases and regulates cell metabolism in response to several nutritional signals, such as nitrogen and carbon source [38]. Sch9p shares many targets with PKA and TORC1, and different interactions between these pathways, either cooperating or antagonizing, have been described [15]. Data from Figure 5D show that *sch9Δ* aa-starved cells underwent increased cell death upon transfer to water plus NH_4_^+^ and that the lack of Sch9p reduced survival after cells were transferred to water. These results suggest that pathways regulated by Sch9p are important for survival under these conditions

To evaluate the dependence of PKA activation on Sch9p, Tor1p, and Mep2p, trehalase activity was measured in aa-starved cells of the corresponding deletion mutants. Trehalase activity was similar in all strains before or after transfer to water. However, in the presence of NH_4_^+^, trehalase activity decreased in *tor1Δ* and in *sch9Δ* cells and was almost completely undetectable in the latter strain (Figure 5A). These results establish that NH_4_^+^ signalling to PKA requires Tor1p and Sch9p. However, the opposite cell death phenotypes of *sch9Δ* compared to *tor1Δ* and *tpk1Δ* cells observed in aa-starved cells in the presence of NH_4_^+^ suggest that the role of Sch9p in the process is essentially independent of the TOR-PKA pathway.

Hog1p is a kinase that regulates and is regulated by Sch9p and mediates stress response independently of PKA and TOR pathways [39]. To assess whether Hog1p might play a role in resistance to the toxic effects of NH_4_^+^ mediated by Sch9p, we examined the effects of NH_4_^+^ in a *hog1Δ* strain. Like *sch9Δ* cells, *hog1Δ* strains were more sensitive to the toxic effects of NH_4_^+^, which suggests that Sch9p may be signaling Hog1p to mediate increased resistance.

### Metabolism of NH_4_^+^ is not required for NH_4_^+^-induced cell death

**Figure 6:**
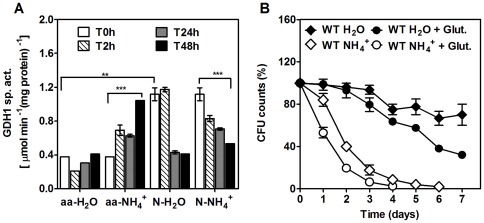
Ammonium-induced loss of cell viability of *S. cerevisiae* does not depend on its metabolization. (A) Glutamate dehydrogenase (GDH1) activity of aa-starved (aa-H_2_O and aa-NH_4_^+^) and N-starved cells (N-H_2_O and N-NH_4_^+^), before transferred to water (T0h) and after 2, 24 and 48 hours in water or water with 0.5% (NH_4_)_2_SO_4_. (B) Survival of wild-type aa-starved cells, in water or water with 0.5% (NH_4_)_2_SO_4_, supplemented or not with glutamate (5 mg/ml). In all the cultures, starting cell density was about 3.8×10^7^ cells/ml and the initial pH was adjusted to 7.0. Values are means ± SEM (n = 3–4). (A) ***P<0.001; **P<0.01 (B) *P*<0.05 (H_2_O *vs* H_2_O+Glut.). Statistical analysis was performed by two-way ANOVA.

The role of Mep2p in signaling PKA activation in response to NH_4_^+^ in nitrogen starvation medium is not dependent on the metabolism of NH_4_^+^ [28]. Therefore, we next asked whether NH_4_^+^ toxicity that leads to a reduction in CLS under our experimental conditions might be signaled directly by NH_4_^+^ or perhaps requires that it be metabolized. In yeasts, the first step of NH_4_^+^ assimilation is mediated by NADPH-dependent glutamate dehydrogenase, which converts α-ketoglutarate to glutamate, which can be further metabolized to glutamine by glutamine synthetase. Glutamine synthetase activity was higher in N-starved cells than in aa-starved cells, indicating that the activity of this enzyme was not related to the higher toxicity of NH_4_^+^ (Table S1). We also tested the effect of NH_4_^+^ in both aa-starved and N-starved cells in the presence of the glutamine synthetase inhibitor methionine sulfoximine. No significant differences in loss of cell viability were observed (Figure S6A and S6B), further supporting the hypothesis that the toxic effect of NH_4_^+^ does not require that it be metabolized. Glutamate dehydrogenase activity at T_0_ was higher in N-starved cells than in aa-starved cells, but incubation in water with or without NH_4_^+^ led to a decrease in its activity (Figure 6A). In contrast, glutamate dehydrogenase activity increased approximately 3-fold in aa-starved cells incubated in the presence of NH_4_^+^. We asked whether α-ketoglutarate depletion or glutamate accumulation, which might result from the higher glutamate dehydrogenase activity, could be the cause of NH_4_^+^ toxicity. Adding α-ketoglutarate to the medium did not alter the toxic effects of NH_4_^+^ (Figure S6C), whereas adding glutamate resulted in more rapid loss in cell viability, even in the absence of NH_4_^+^ (Figure 6B). Furthermore, the non-metabolizable NH_4_^+^ analogue methylamine also induced cell death in aa- but not N-starved cells (Figure S6D). In agreement with these results, the NH_4_^+^ toxicity observed in SC media cultures (Fig. 1) was also not associated with a significant NH_4_^+^metabolization, as depicted from the levels of (NH_4_)_2_SO_4_ along time (Fig. S1A).

Taken together, these results suggest that although glutamate could play a role in NH_4_^+^-induced cell death to some extent, NH_4_^+^-induced shortening of CLS does not appear to require that it be metabolized.

## Discussion

Our studies demonstrate that at high concentrations, NH_4_^+^, which is a commonly employed source of nitrogen in laboratory yeast cultures, induces cell death in association with a reduction in CLS. The toxic effects of NH_4_^+^ correlate with NH_4_^+^ concentration in the culture medium and were enhanced in cells starved for auxotrophy-complementing amino acids. Addition of NH_4_^+^ to cultures after they were transferred to water reduced cell survival, indicating that NH_4_^+^ alone could also induce loss of cell viability as observed in culture media. Overall the results suggest NH_4_^+^ is a factor accounting for the loss of cell viability in aging cells. Although some of the toxic effects of NH_4_^+^ were accompanied by markers for apoptosis, NH_4_^+^ -induced cell death was predominantly necrotic at later time points. Our data suggest that NH_4_^+^ causes an initial apoptotic cell death followed by a fast secondary necrosis. Necrosis due to ATP depletion has been reported in other cell death scenarios, namely in tumor cells under metabolic stress [40]. This appears to be the case in NH_4_^+^-induced necrosis, since ATP depletion was observed in cells incubated in water with NH_4_^+^ (Figure S3B), which might block ATP-dependent apoptosis and thus trigger necrosis. The results obtained with the deletion mutant *rim13Δ* point to a protective function of the protease calpain in this cell death process.

As discussed in the Introduction, cells in G0 acquire a variety of characteristics including induction of autophagy [8], [12]. Previous studies showed that cells starved for auxotrophic amino acid markers in otherwise complete medium fail to properly arrest in G0 [41]. In accordance, aa-starved cells in our study also do not seem arrested in G0 (indicated by a failure to induce autophagy). It should be noted that this failure to induce autophagy by aa-starved cells was sustained when cells were transferred to water containing NH_4_^+^ in the absence of other nutrients. This is in contrast with that observed in G0 arrested N-starved cells transferred to water where NH_4_^+^ could not activate PKA or inhibit autophagy. Although autophagy was inhibited by NH_4_^+^ in aa-starved cells, inhibition of autophagy by deletion of *ATG8* did not induce the NH_4_^+^ sensitivity phenotype in N-starved cells, suggesting that autophagy inhibition is not responsible for the loss of cell viability and shorter CLS induced by NH_4_. We also assessed whether activation of PKA could be inducing replication stress, a mechanism responsible for cell aging under different conditions [42]. This could be the case, at least to some extent, since there was a slight increase in the number of budded cells (evaluated by bright field microscopy) for aa-starved (16%) conditions relative to the control N-starved cells (8%).

In contrast to what has been described for aging cells that reach stationary phase due to carbon limitation [43], we observed that autophagy mutants did not exhibit increased cell death after they were transferred to water, indicating that autophagy is not a key player in cell survival in water when the cells were previously starved for amino acids or nitrogen. It was recently shown that *ATG* genes are important for removing ROS and for maintaining mtDNA and mitochondrial function [44]. This may explain the lack of dependence of cell survival on autophagy in our experimental conditions, as the production of ROS was relatively low. Hence, the cell physiological state resulting from different culture conditions influences not only life span extension [2], but also the cellular processes essential for its regulation.

In yeasts, the TOR, Sch9p and PKA pathways are key players in the regulation of CLS [6]. In our study, activation of PKA correlates with sensitivity to NH_4_^+^, which is partially suppressed by deletion of *RAS2,* indicating the RAS/Cyr1/PKA pathway is involved in this process. Partial, but not complete, suppression of these effects when *RAS2* is deleted suggests either that the second RAS isoform (*RAS1*) also participates in NH_4_^+^-induced PKA activation or the existence of two pathways responsible for NH_4_^+^ toxicity, one that depends on RAS/Cyr1/PKA and one that is independent of this pathway. In nitrogen starvation medium, addition of NH_4_^+^ directly signals PKA activation through Mep2p and does not depend on its metabolization [28]. Our results show that Mep2p is involved in NH_4_^+^-induced death but does not appear to have a major role in PKA activation. Still, although glutamate could somewhat mediate the effect of NH_4_^+^, CLS shortening also seemed to be directly signalled by NH_4_^+^, as it was not dependent on its metabolization to either glutamate or glutamine.

The deletion of *TPK1,* but not of *TPK2* or *TPK3,* encoding the other two PKA isoforms, significantly reverted the NH_4_^+^-induced death and shorter CLS. These results suggest that different programmed cell death processes can be regulated by distinct PKA isoforms, since Tpk3p has been reported to regulate apoptosis induced by actin stabilization [45]. Our data are also in agreement with previous results showing that CLS extension of glucose-growth limited stationary phase cells depends on PKA inactivation [46]. Our results indicate that PKA inactivation cannot extend cell survival time in the absence of Sch9p, since we observed that *SCH9* deletion abolishes PKA activation in response to NH_4_^+^, but does not rescue the shortening in CLS induced by NH_4_^+^. Furthermore, the phenotype of aa-starved *sch9Δ* cells in the presence of NH_4_^+^ was the opposite of that of *tor1Δ* and *tpk1Δ*, suggesting that the role of Sch9p in the process is essentially independent of the TOR-PKA pathway mediated by a TORC1-Sch9 effector branch. Instead, the two pathways likely regulate their downstream targets that are involved in NH_4_^+^-induced cell death in an opposing manner. Consistent with this possibility, it was reported that Sch9p positively regulates many stress-response genes and genes involved in mitochondrial function, whereas the same classes of genes are inhibited by the TOR1C pathway [15]. Data suggest that Sch9p may mediate survival in response to NH_4_^+^ through activation of Hog1p, the yeast closest homolog to p-38 and c-JNK of mammalian cells [47]. Previous reports have shown that *sch9Δ* yeast cells exhibit a longer CLS compared to wild type cells, when aging in SC medium or after transfer from this medium to water [46], [48]. Differences in strain background and/or in culture conditions may account for the discrepancy in results [46], [48]–[50]. Supporting this explanation it was also previously reported that *SCH9* deletion shortened the CLS survival of S288c-based strains (as is the case of BY4742 strain used in the present work) pregrown on glycerol [1].

**Figure 7:**
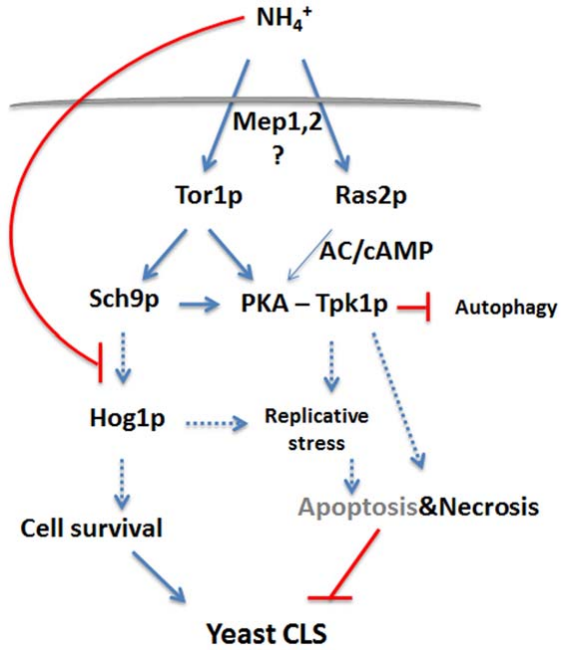
Proposed mechanism for the regulation of cell death associated to CLS shortening induced by ammonium in amino acid-starved yeast cells. NH_4_^+^ activates PKA through both RAS and TOR/Sch9p and leads to cell death increase with predominant necrotic features associated to ATP depletion. Sch9p is mediating survival in response to NH_4_^+^ possibly through activation of Hog1p.

In conclusion, here we have shown that NH_4_^+^ induces cell death in aging yeast in association with a reduction in CLS, both of which are positively correlated with NH_4_^+^ concentration in the culture medium. Furthermore, these effects are enhanced in cells starved for auxotrophy-complementing amino acids. As for the mechanism involved (Figure 7), the results indicate that in aa-starved cells NH_4_^+^ activates PKA through both RAS and TOR/Sch9p signalling cascades and leads to cell death increase with predominant necrotic features. The mediation of NH_4_^+^ effects seems to involve the NH_4_^+^ permeases Mep2 and (to a lesser extent) Mep1 as sensors. Sch9p is also mediating survival in response to NH_4_^+^ possibly through activation of Hog1p. NH_4_^+^ action on both pathways culminates in the shortening of CLS.

As discussed in the introduction, NH_4_^+^ is toxic for mammals, and NH_4_^+^-induced cell death is involved in different human disorders that are accompanied by hyperammonemia, such as hepatic encephalopathy [21]. Here we extensively characterized for the first time a cell death process induced by NH_4_^+^ in yeast cells. This process shares common features with NH_4_^+^-induced cell death in brain cells. A better understanding of NH_4_^+^-induced cell death in the yeast cell model can help clarify controversial issues on NH_4_^+^ toxicity associated to hyperamonemia that are not easy to examine in more complex models. Our results show that the effect of NH_4_^+^ is not due to different levels of NH_4_^+^ metabolization, an open question for brain cells, but relies on the over-activation of PKA and the TOR pathway and inhibition of Sch9p (yeast closest homolog of mammalian Akt and S6K). On the other hand, the mitogen activated protein kinase (MAPK) Hog1p was associated with higher cell viability in the presence of NH_4_^+^ similarly to what was found for its human homolog p38 that mediates endogenous cell protection in response to ammonium in astrocytes [51]. Also, we observed that NH_4_^+^ toxicity is higher in non-arrested cells, which is consistent with the observation that hyperammonemia presents with much more severe consequences in the developing brain of newborns or infants than in adulthood. Furthermore, our data link NH_4_^+^ toxicity to amino acid limitation, a situation that can also be present in hyperammonemic patients, who are often on dietary protein restriction [52]. Further experiments will be necessary to establish whether over-activation of TOR and PKA pathways and inhibition of Sch9p is a widely conserved mechanism in NH_4_^+^ toxicity and induction of cell death. We believe that our model can be useful in the elucidation of conserved mechanisms and pathways of NH_4_^+^-induced cell death and in identification of therapeutic targets for diseases associated with hyperammonemia. Deprivation of essential amino acids has been employed as a strategy in cancer therapy, but resistance has often been found. Our results establishing that NH_4_^+^ can stimulate cell death in amino acid-deprived cells and suggests that *S. cerevisiae* might serve as useful model for the identification of signaling pathways for this disease. Furthermore, our finding that NH_4_^+^ decreases cell survival during aging through the regulation of the evolutionary conserved pathways PKA and TOR also enriches our understanding of longevity regulation in multicellular organisms.

## Materials and Methods

### Strains and growth conditions

*Saccharomyces cerevisiae* strain BY4742 (*MAT*a *his3Δ*1 *leu2Δ*0 *lys2Δ*0 *ura3*Δ0) (EUROSCARF, Frankfurt, Germany) and the respective knockouts in *YAC1*, *AIF1*, *RIM13*, *RAS2*, *CPR3*, *ATG8*, *SCH9*, *MEP1, MEP2, TPK1, TPK2, TPK3* and *TOR1* genes, were used. For experiments with stationary phase cells with or without restriction of auxotrophy-complementing amino acids, cells were cultured at 26°C, 150 rpm, for 72 hours, in defined minimal medium (SC medium) containing 0.17% yeast nitrogen base without amino acids and without ammonium sulphate (Difco, BD), 2% D-glucose; supplemented with 0.01%, 0.1%, 0.5% or 1% ammonium sulphate, and with low (10 mg/l histidine, 10 mg/l lysine, 60 mg/l leucine and 100 mg/l uracil) or high (50 mg/l histidine, 50 mg/l lysine, 300 mg/l leucine and 100 mg/l uracil) concentrations of essential amino acids. After 72 hours, cells were collected by centrifugation and: A) resuspended in growth medium (exhausted medium without adjusting pH - pH 2.9) with a cell density of about 3.8×10 ^7^cells/ml, B) resuspended in growth medium (exhausted medium) with a cell density of about 3.8×10 ^7^cells/ml, with pH adjusted to 7.0.; C) resuspended in water (pH 7.0), after being washed three times, at cell density of about 3.8×10^7^ cells/ml; D) resuspended in water with ammonium sulphate (0.5% or 1%, pH 7.0.), after being washed three times, at cell density of about 3.8×10^7^ cells/ml. Viability of stationary 3 day old cultures was considered to be 100% of survival and this was considered day 0 of the experiment. pH 7.0 was maintained throughout the experiment in cultures with adjusted pH. For experiments with aa- and N-starved cells, cells were first cultured at 26°C and 150 rpm, in the defined minimal medium described above, supplemented with 0.5% ammonium sulphate, appropriate amino acids and base (50 mg/l histidine, 50 mg/l lysine, 300 mg/l leucine and 100 mg/l uracil) and 2% D-glucose, to exponential phase (OD_600_ = 1.0–1.5). These cells were harvested and resuspended in nitrogen-starvation medium (N-) containing 4% glucose and 0.17% yeast nitrogen base without amino acids and ammonium sulphate, or in amino acid-starvation medium (aa-) containing the same components as N-starvation medium plus 0.5% ammonium sulphate. After 24 hours, cells were collected by centrifugation and: A) resuspended in starvation medium (N- or aa-) with a cell density of about 3.8×10^7^cells/ml, without adjusting pH (pH 2.7); B) resuspended in starvation medium (N- or aa-) with a cell density of about 3.8×10^7^cells/ml, with pH adjusted to 7.0; C) resuspended in water (pH 7.0), after being washed three times, at cell density of about 3.8×10^7^ cells/ml; D) resuspended in water with ammonium sulphate (0.5%, pH 7.0.), after being washed three times, at cell density of about 3.8×10^7^ cells/ml. Viability of 24 hours starved cultures was considered to be 100% of survival and this was considered day 0 of the experiment. pH 7.0 was maintained throughout the experiment in cultures with adjusted pH. Cell viability was assessed by Colony Forming Units (CFU) at day 0 (100% viability) and in subsequent days, as indicated, of culture aliquots incubated for 2 days at 30°C on YEPD agar plates. BY4742 strain was transformed with plasmids pUG35 and pUG35- *NHP6A-EGFP* [17], kindly provided by Dr. Frank Madeo (University of Gratz, Austria), and was cultured as described above for aa-starved cells, in medium lacking uracil. The methodology of aging experiments with stationary phase cells and with amino acid (aa)- and nitrogen(N)-starved cells is schematically represented in Figure S1.

### Ammonium and ATP Determination

Ammonium in the culture media was quantified by Dr. José Coutinho (University of Trás-os-Montes e Alto Douro, Portugal) as previously described [53].

ATP measurements were performed according to [54]. Briefly, cells were collected by centrifugation and the pellet was frozen with liquid nitrogen and stored at −80°C. For the ATP assay, the pellet was mixed with 200 µl of 5% TCA and vortexed for one minute, twice, with one minute interval on ice. This mix was centrifuged for one minute, at 4°C, and 10 µl of the supernatant were added to 990 µl of reaction buffer (25 mM HEPES, 2 mM EDTA, pH 7.75). 100 µl of this mixture was added to 100 µl of Enliten Luciferin/Luciferase Reagent (Promega) and luminescence was measured on a ThermoScientific Fluoroskan Ascent FL.

### Measurements of cell death markers

For the detection of chromatin changes, cells were stained with 4,6-diamido-2-phenyl-indole (DAPI, Sigma) according to [55]. DNA strand breaks were assessed by TUNEL with the ‘In Situ Cell Death Detection Kit, Fluorescein’ (Roche Applied Science) as described previously [55]. In both assays, and also for the nuclear release of the necrotic marker Nhp6Ap–EGFP, cells were visualized by epifluorescence in a Leica Microsystems DM-5000B microscope, at least 300 cells of three independent experiments being evaluated, with appropriate filter settings using a 100×/1.3 oil-immersion objective. Images were acquired with a Leica DCF350FX digital camera and processed with LAS AF Leica Microsystems software. To measure DNA content, cells were stained with SYBR Green I as described [56] and staining was assessed by flow cytometry. Plasma membrane integrity was assessed by incubating cells with 5 mg ml^−1^ propidium iodide (PI) (Molecular Probes, Eugene, OR) for 10 minutes at room temperature followed by flow cytometry measurements of PI-stained cells. Intracellular reactive oxygen species were detected by dihydrorhodamine (DHR)-123 staining or dihydroethidium (DHE) (Molecular Probes). For DHR, cells were incubated with 15 mg/mL of DHR-123 for 90 min at 30°C in the dark, washed in PBS and evaluated by flow cytometry. For DHE, cells were incubated with 5 µM and after incubation for 10 min at 30°C cells were washed once with PBS and evaluated by flow cytometry. Phosphatidylserine exposure was detected by FITC Annexin-V (BD Pharmingen) as described previously [55]. Briefly, cell walls were digested with 3% (v/v) glusulase (NEE-154 Glusulase; Perkinelmer) and 7 U/ml lyticase (Sigma) for 40 minutes, at 28°C. For intracellular calcium measurements, cells previously washed with PBS were stained with 10 µM FLuo3 AM (Molecular Probes, Eugene, OR) for 2 hours at 30°C in the dark, subsequently washed in PBS and assessed by flow cytometry. Flow cytometry analysis of the above experiments was performed in an Epics® XL™ (Beckman Coulter) flow cytometer, equipped with an argon ion laser emitting a 488 nm beam at 15 mW. The green fluorescence was collected through a 488-nm blocking filter, a 550-nmlong-pass dichroic and a525-nm bandpass. Red fluorescence was collected through a 488-nm blocking filter, a 590-nmlong-pass dichroic and a620-nm bandpass. Thirty thousand cells per sample were analyzed. Positive controls for apoptosis involved treatment of cells with 160 mM acetic acid for 200 minutes, at pH 3 and 3 mM H_2_O_2_ at pH 3. For the necrotic marker Nhp6Ap–EGFP, no nuclear release was observed in the presence of 3 mM H_2_O_2_.

### Treatments

Methionine sulfoximine (MSX,Sigma), an irreversible inhibitor of glutamine synthetase, was dissolved in sterile water at a concentration of 100 mM and stored at 4°C. MSX was added to water (pH 7.0), and water with ammonium sulphate (0.5%, pH 7.0), at the concentration of 1 mM. Wortmannin (Sigma), a PI3K inhibitor, was added to water (pH 7.0), and water with ammonium sulphate (0.5%, pH 7.0), at the concentration of 6 µM or 23 µM. Glutamate was added to water (pH 7.0), and water with ammonium sulphate (0.5%, pH 7.0), at the concentration of 5 mg/ml. Adenosine 3′,5′-cyclic monophosphate (cAMP, Sigma) was added to aa-starvation or N-starvation medium or to water (pH 7.0), and water with ammonium sulphate (0.5%, pH 7.0), at the concentration of 4 mM. α-Ketoglutaric acid potassium salt (Sigma) was added to water (pH 7.0), and water with ammonium sulphate (0.5%, pH 7.0), at the concentration of 5 mg/ml. Cyclosporin A (Sigma) was added to water (pH 7.0), and water with ammonium sulphate (0.5%, pH 7.0.), at the concentration of 120 µg/ml.

### Western Blot analysis

Western blot analysis was performed according to [57]. For Atg8p and Pgk1p detection, rabbit polyclonal anti-Aut7 (1∶200; Santa Cruz Biotech) and mouse monoclonal anti-PGK1 (1∶5000; Molecular Probes) were used, respectively, followed by Peroxidase-AffiniPure Goat Anti-Rabbit IgG (1∶10000; Jackson ImmunoResearch).

### Enzyme assays

Glutamine synthetase (Gs) assay was performed according to [58]. Glutamate dehydrogenase activity was determined according to [59]. Briefly, cell extracts were prepared by adding to the cell pellet a roughly equal volume of 0.5 mm diameter glass beads in the presence of 0.1 M potassium phosphate buffer (pH 6.0), followed by vigorous mixing during 1 minute intervals interspersed with periods of cooling in ice. The NADP-dependent GDH activity was determined by following the disappearance of NADPH at 340 nm. Trehalase activity was determined according to [60]. Briefly, crude enzyme extracts were obtained by ressuspending the cell pellet in ice-cold 50 mM MES/KOH buffer (pH 7.0) containing 50 µM CaCl_2_, and adding a roughly equal volume of 0.5 mm diameter glass, followed by vigorous mixing during 1 minute intervals interspersed with periods of cooling in ice. The extracts were then dialyzed overnight at 4°C in a dialysis cellulose membrane (Cellu Sep H1, Orange). The dialyzed extract was then used to assess trehalase activity by measuring the liberated glucose with glucose oxidase assay (GOD, Roche).

## Supporting Information

Figure S1 **Ammonium levels in medium during culture of** ***S. cerevisiae*** **with insufficient supply of amino acids, and cell death induced by NH_4_OH or by (NH_4_)_2_SO_4_ in the presence of increased potassium concentration.** (A) Quantification of (NH_4_)_2_SO_4_ in SC medium supplemented with low concentrations of auxotrophy-complementing amino acids and 0.5% (NH_4_)_2_SO_4_, during culture of wild-type cells; day −3 represents the day of culture inoculation and day zero represents the beginning of aging experiments. (B) Survival of wild-type (WT) aa-starved cells, in water or water with 0.5% NH_4_OH. (C) Survival of wild-type (WT) aa-starved cells, in water, water with 0.5% (NH_4_)_2_SO_4_ and water with 0.5% (NH_4_)_2_SO_4_ supplemented with 13 mM K_2_SO_4_. Values are means ± SEM (n = 3).
(TIF)

Figure S2 **Scheme of the methodology used.** (A) experiments with the stationary phase cells and (B) experiments with aa- and N-starved cells.
(TIF)

Figure S3 **Effect of ammonium on the cell cycle and ATP content.** (A) Cell cycle histograms of aa-starved and N-starved *S. cerevisiae* wild-type cells at day 0 and day 5 upon transfer to water or water with 0.5% (NH_4_)_2_SO_4_, after a 24 hour period in starvation (aa- and N-) media. (B) ATP content of aa-starved cells (day 0, 1, 2 and 3) upon transfer to water or water with 0.5% (NH_4_)_2_SO_4_. Values are means ± SEM (n = 3). (B) ***P<0.001 (T0 *vs* T1,2 and 3).
(TIF)

Figure S4 **Effect of** ***ATG8*** **deletion and of the inhibitors wortmannin and cycloheximide in NH_4_^+^ - induced cell death in** ***S. cerevisiae.*** Survival of wild-type (WT) and *atg8*Δ mutant (A) aa-starved or (C) N-starved cells, in water or water with 0.5% (NH_4_)_2_SO_4_. Survival of WT aa-starved cells, in water or water with 0.5% (NH_4_^+^)_2_SO_4_, supplemented with (B) wortmannin (WN) or (D) cycloheximide (0.01%). Values are means ± SEM (n = 3). (A); (B) and (D) *P*<0.001 (H_2_O *vs* 0.5% (NH_4_)_2_SO_4_). Statistical analysis was performed by two-way ANOVA.
(TIF)

Figure S5 **Loss of cell viability induced by NH_4_^+^ in aa-starved cells in water, of** ***S. cerevisiae*** **wild-type (WT) and mutants deleted in the genes coding for the yeast metacaspase (Yca1), the apoptosis inducing factor (Aif1), mitochondrial cyclophylin (Cpr3) and calpain (Rim13).** Survival of (A) WT and *yca1Δ*, (B) WT and *aif12Δ*, (C) WT and *rim13Δ* and (D) WT and *cpr3Δ* aa-starved cells, in water or water with 0.5% (NH_4_)_2_SO_4_. (E) Survival of WT aa-starved cells, in water or water with 0.5% (NH_4_)_2_SO_4_, supplemented or not with cyclosporine A (CsA) (120 µg/ml). In all the cultures, starting cell density was about 3.8×10^7^cells/ml and the initial pH was adjusted to 7.0. Values are means ± SEM (n = 3–4). (A), (B), (D) and (E) *P*<0.001 (H_2_O *vs* 0.5% (NH_4_^+^)_2_SO_4_); (C) *P*<0.001 (WT 0.5% (NH_4_)_2_SO_4_ *vs rim13Δ* 0.5% (NH_4_^+^)_2_SO_4_). Statistical analysis was performed by two-way ANOVA.
(TIF)

Figure S6 **Metabolism of NH_4_^+^ is not required for NH_4_^+^-induced cell death in** ***S. cerevisiae*** **.** Survival of wild-type (WT) aa-starved cells (A) or N-starved cells (B), in water or water with 0.5% (NH_4_)_2_SO_4_, supplemented with methionine sulfoximine (MSX) (1 mM). (C) Survival of WT aa-starved cells, in water or water with 0.5% (NH_4_)_2_SO_4_, supplemented with α-ketoglutarate (α-KG) (5 mg/ml). (D) Survival of WT aa-starved or N-starved cells, in water or water with 0.5% (NH_4_)_2_SO_4_, supplemented with methylamine (MA) (30 mM). In all the cultures, starting cell density was about 3.8×10^7^cells/ml and the initial pH was adjusted to 7.0. Values are means ± SEM (n = 3–4). (A), (C) and (D) *P*<0.001 (H_2_O *vs* 0.5% (NH_4_)_2_SO_4_). Statistical analysis was performed by two-way ANOVA.
(TIF)

Table S1 **Glutamine synthetase (GS) activity of aa- and N-starved cells of** ***S. cerevisiae*** **before (T0) and after transfer to water or water with 0.5% (NH_4_)_2_SO_4_.**
(DOCX)
