# Supplementary figures and images for: Ammonium Is Toxic for Aging Yeast Cells, Inducing Death and Shortening of the Chronological Lifespan
Source: PLoS One. 2012 May 15;7(5):e37090. doi: 10.1371/journal.pone.0037090 (PMC3352862; doi:10.1371/journal.pone.0037090)

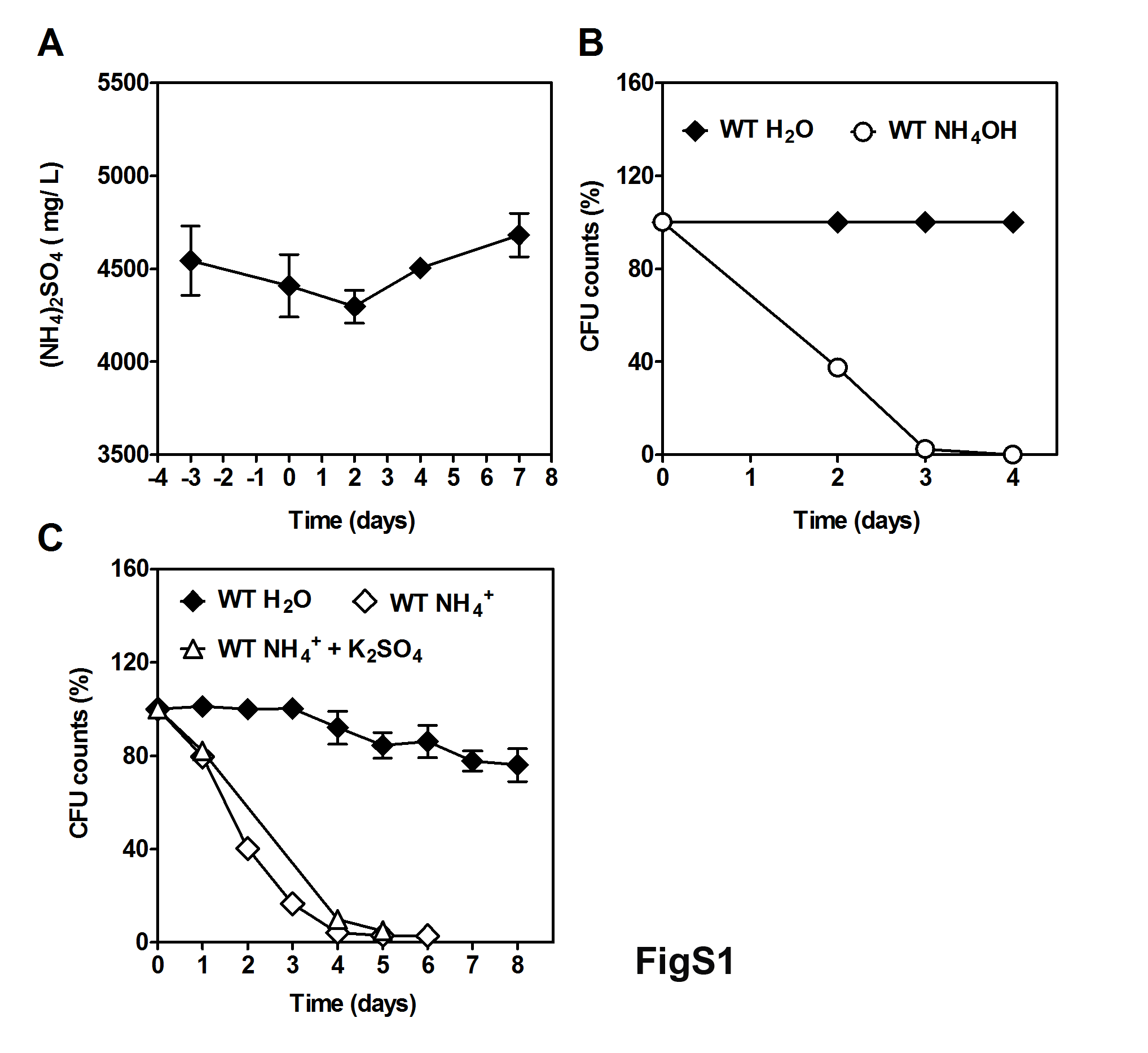

Supplement: Figure S1 — Ammonium levels in medium during culture of S. cerevisiae with insufficient supply of amino acids, and cell death induced by NH4OH or by (NH4)2SO4 in the presence of increased potassium concentration. (A) Quantification of (NH4)2SO4 in SC medium supplemented with low concentrations of auxotrophy-complementing amino acids and 0.5% (NH4)2SO4, during culture of wild-type cells; day −3 represents the day of culture inoculation and day zero represents the beginning of aging experiments. (B) Survival of wild-type (WT) aa-starved cells, in water or water with 0.5% NH4OH. (C) Survival of wild-type (WT) aa-starved cells, in water, water with 0.5% (NH4)2SO4 and water with 0.5% (NH4)2SO4 supplemented with 13 mM K2SO4. Values are means ± SEM (n = 3). (TIF) [file pone.0037090.s001.tif]

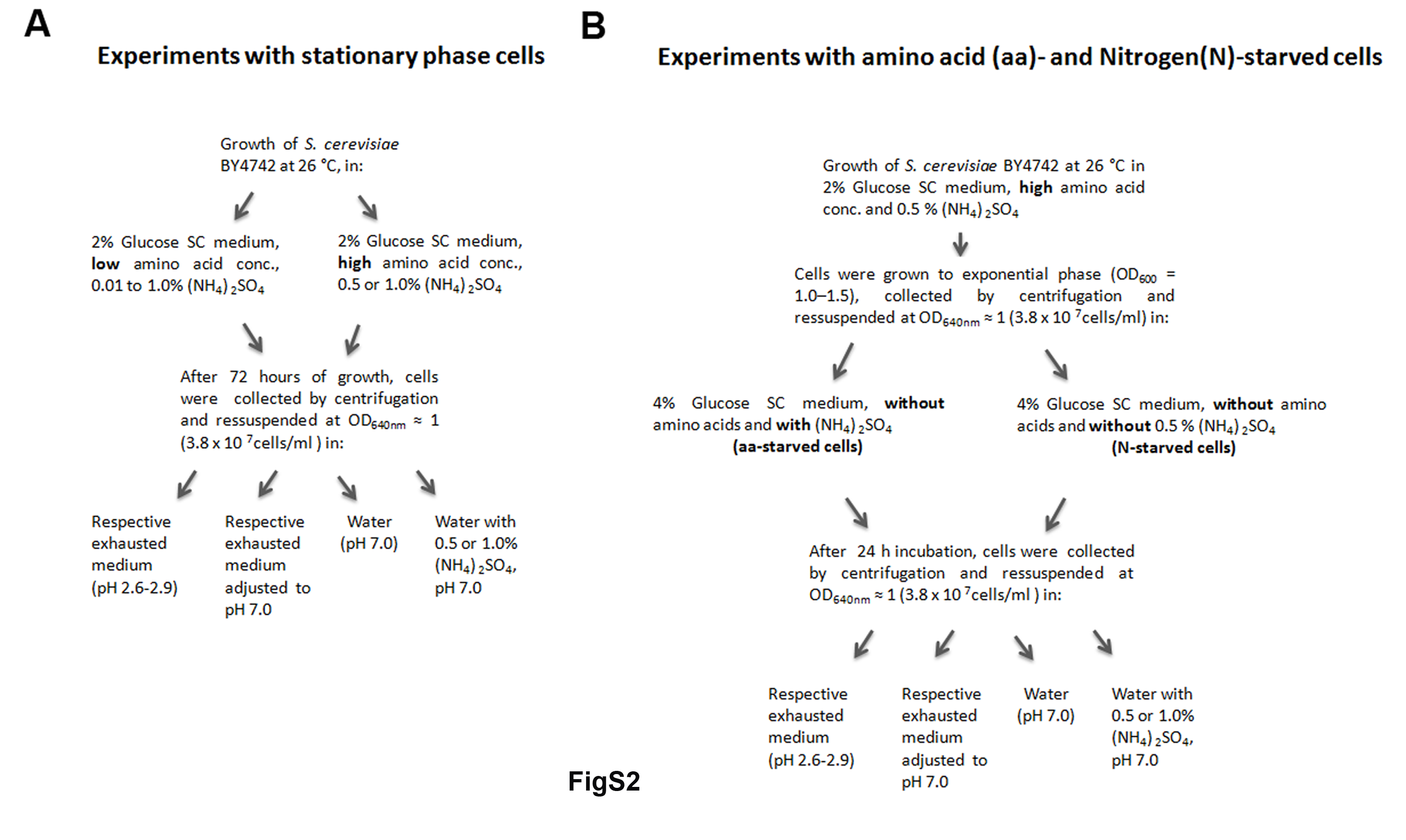

Supplement: Figure S2 — Scheme of the methodology used. (A) experiments with the stationary phase cells and (B) experiments with aa- and N-starved cells. (TIF) [file pone.0037090.s002.tif]

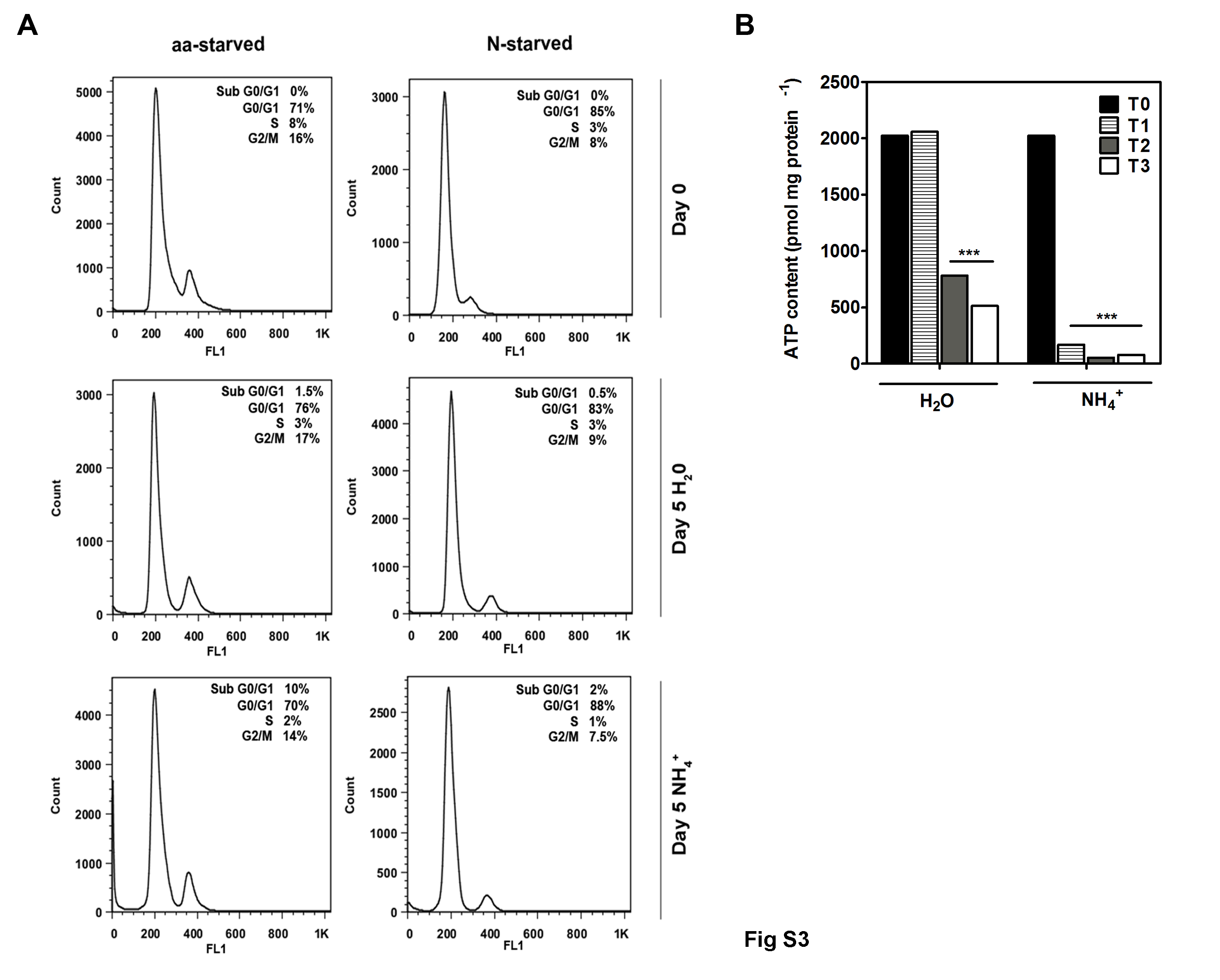

Supplement: Figure S3 — Effect of ammonium on the cell cycle and ATP content. (A) Cell cycle histograms of aa-starved and N-starved S. cerevisiae wild-type cells at day 0 and day 5 upon transfer to water or water with 0.5% (NH4)2SO4, after a 24 hour period in starvation (aa- and N-) media. (B) ATP content of aa-starved cells (day 0, 1, 2 and 3) upon transfer to water or water with 0.5% (NH4)2SO4. Values are means ± SEM (n = 3). (B) ***P<0.001 (T0 vs T1,2 and 3). (TIF) [file pone.0037090.s003.tif]

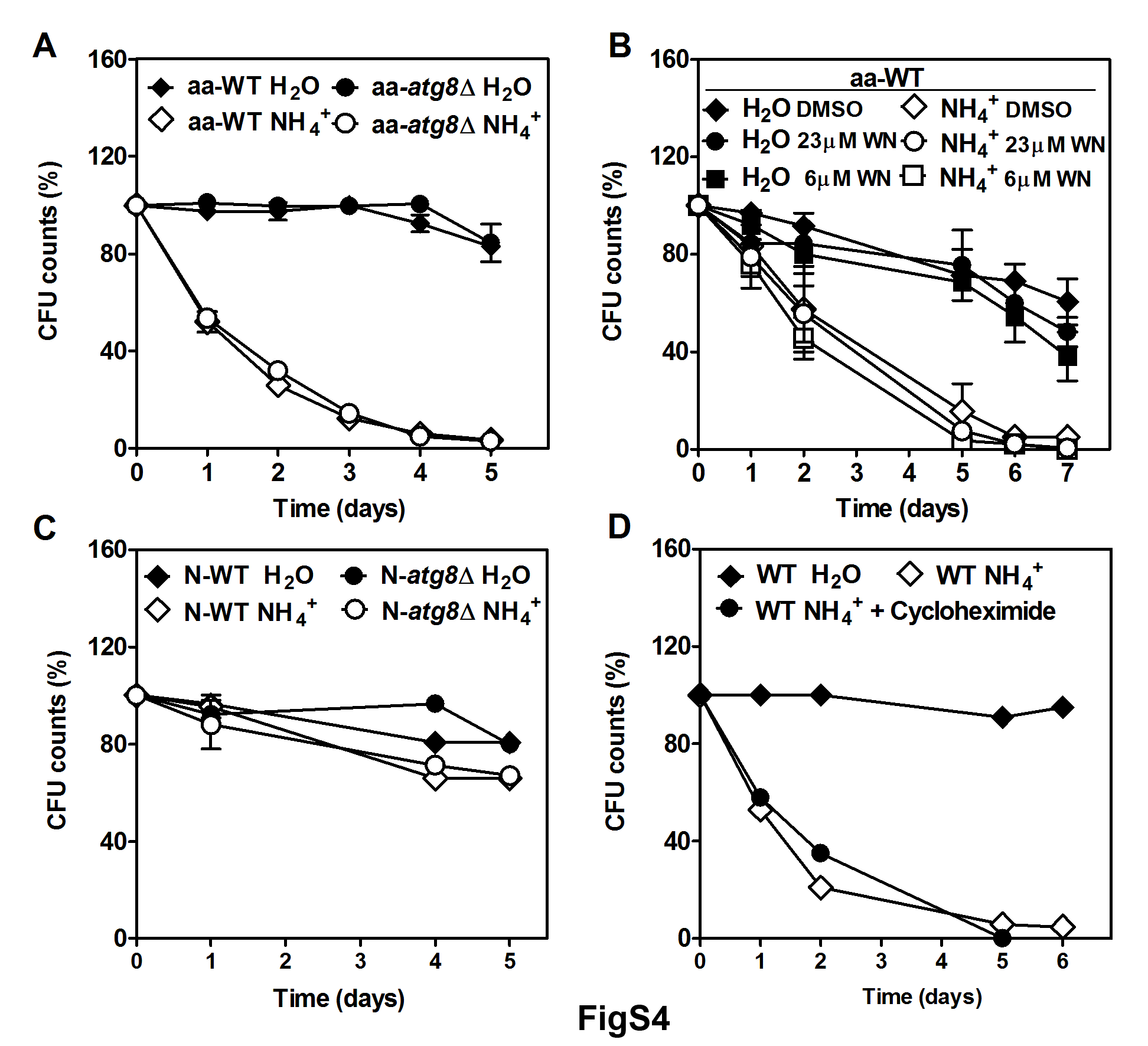

Supplement: Figure S4 — Effect of ATG8 deletion and of the inhibitors wortmannin and cycloheximide in NH4+ - induced cell death in S. cerevisiae. Survival of wild-type (WT) and atg8Δ mutant (A) aa-starved or (C) N-starved cells, in water or water with 0.5% (NH4)2SO4. Survival of WT aa-starved cells, in water or water with 0.5% (NH4 +)2SO4, supplemented with (B) wortmannin (WN) or (D) cycloheximide (0.01%). Values are means ± SEM (n = 3). (A); (B) and (D) P<0.001 (H2O vs 0.5% (NH4)2SO4). Statistical analysis was performed by two-way ANOVA. (TIF) [file pone.0037090.s004.tif]

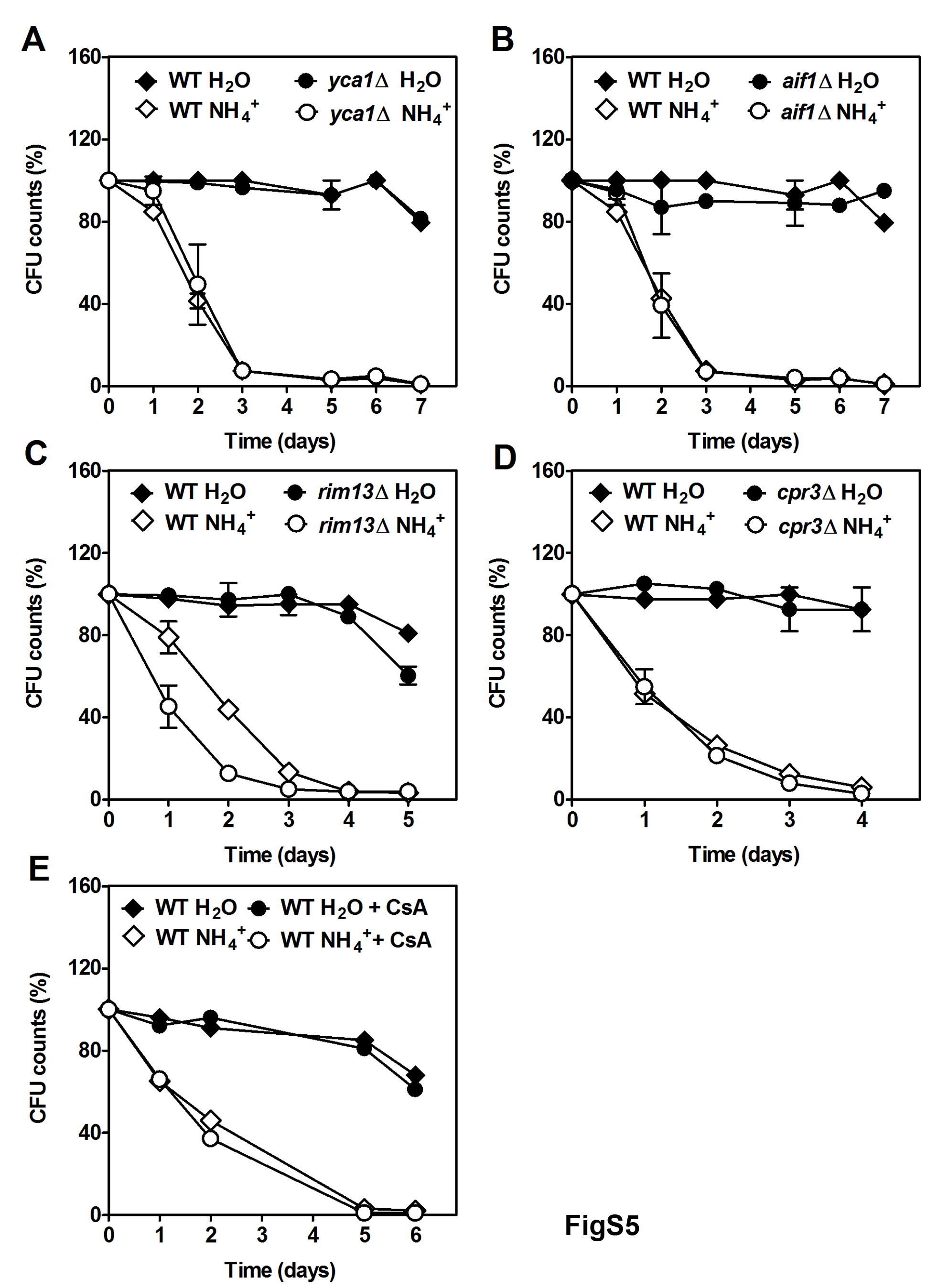

Supplement: Figure S5 — Loss of cell viability induced by NH4+ in aa-starved cells in water, of S. cerevisiae wild-type (WT) and mutants deleted in the genes coding for the yeast metacaspase (Yca1), the apoptosis inducing factor (Aif1), mitochondrial cyclophylin (Cpr3) and calpain (Rim13). Survival of (A) WT and yca1Δ, (B) WT and aif12Δ, (C) WT and rim13Δ and (D) WT and cpr3Δ aa-starved cells, in water or water with 0.5% (NH4)2SO4. (E) Survival of WT aa-starved cells, in water or water with 0.5% (NH4)2SO4, supplemented or not with cyclosporine A (CsA) (120 µg/ml). In all the cultures, starting cell density was about 3.8×10^7^cells/ml and the initial pH was adjusted to 7.0. Values are means ± SEM (n = 3–4). (A), (B), (D) and (E) P<0.001 (H2O vs 0.5% (NH4 +)2SO4); (C) P<0.001 (WT 0.5% (NH4)2SO4 vs rim13Δ 0.5% (NH4 +)2SO4). Statistical analysis was performed by two-way ANOVA. (TIF) [file pone.0037090.s005.tif]

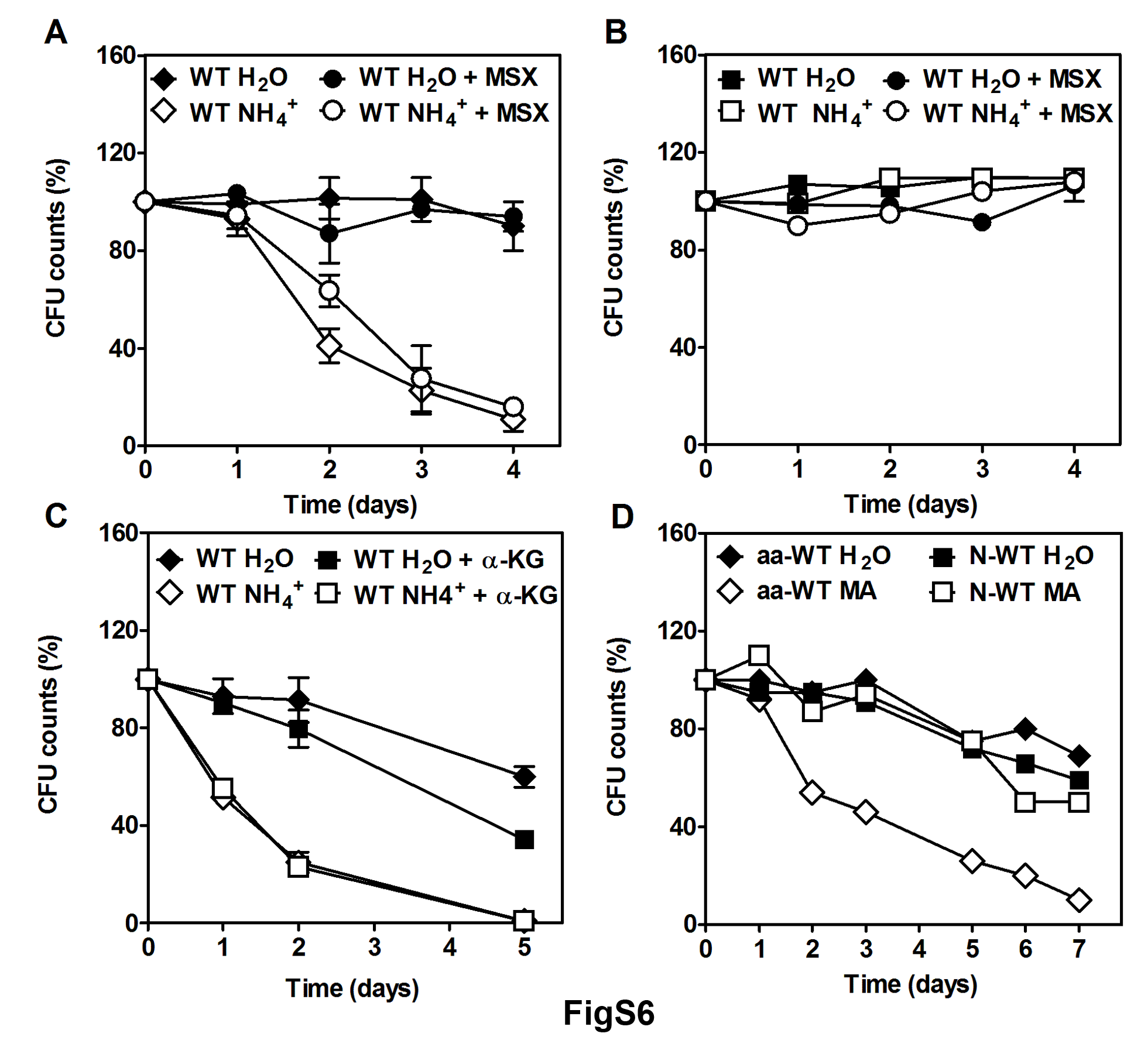

Supplement: Figure S6 — Metabolism of NH4+ is not required for NH4+-induced cell death in S. cerevisiae . Survival of wild-type (WT) aa-starved cells (A) or N-starved cells (B), in water or water with 0.5% (NH4)2SO4, supplemented with methionine sulfoximine (MSX) (1 mM). (C) Survival of WT aa-starved cells, in water or water with 0.5% (NH4)2SO4, supplemented with α-ketoglutarate (α-KG) (5 mg/ml). (D) Survival of WT aa-starved or N-starved cells, in water or water with 0.5% (NH4)2SO4, supplemented with methylamine (MA) (30 mM). In all the cultures, starting cell density was about 3.8×10^7^cells/ml and the initial pH was adjusted to 7.0. Values are means ± SEM (n = 3–4). (A), (C) and (D) P<0.001 (H2O vs 0.5% (NH4)2SO4). Statistical analysis was performed by two-way ANOVA. (TIF) [file pone.0037090.s006.tif]
