# Supplementary material for: Ammonium Is Toxic for Aging Yeast Cells, Inducing Death and Shortening of the Chronological Lifespan
Source: PLoS One. 2012 May 15;7(5):e37090. doi: 10.1371/journal.pone.0037090 (PMC3352862; doi:10.1371/journal.pone.0037090)
Supplement: Table S1 — Glutamine synthetase (GS) activity of aa- and N-starved cells of S. cerevisiae before (T0) and after transfer to water or water with 0.5% (NH4)2SO4. (DOCX) [file pone.0037090.s007.docx]

Table S1.

| Time  (Hours) | sp. act.*  [ µmol min^-1^(mg protein)^-1^] | | | |
| --- | --- | --- | --- | --- |
|  | aa-starved | | N-starved | |
| T0 | 0.062 ± 0.021 | | 0.137 ± 0.009 | |
|  | H_2_O | NH_4_^+^ | H_2_O | NH_4_^+^ |
| T2 | 0.054 ± 0.025 | 0.040 ± 0.011 | 0.092 ± 0.016 | 0.068 ± 0.005 |
| T24 | 0.030 ± 0.011 | 0.019 ± 0.021 | 0.123 ± 0.020 | 0.056 ± 0.004 |
| T48 | 0.008 ± 0.001 | 0.008 ± 0.001 | 0.089 ± 0.012 | 0.031 ± 0.010 |

* Data are presented as mean of three biological replicates with SD.
